# Activation of the Hypoglossal to Tongue Musculature Motor Pathway by Remote Control

**DOI:** 10.1038/srep45860

**Published:** 2017-04-06

**Authors:** Garret A. Horton, Jimmy J. Fraigne, Zoltan A. Torontali, Matthew B. Snow, Jennifer L. Lapierre, Hattie Liu, Gaspard Montandon, John H. Peever, Richard L. Horner

**Affiliations:** 1 Department of Physiology, University of Toronto, Toronto, M5S 1A8, Canada; 2 Department of Cell and Systems Biology, University of Toronto, Toronto, M5S 3G5, Canada; 3 Department of Medicine, University of Toronto, Toronto, M5S 1A8, Canada

## Abstract

Reduced tongue muscle tone precipitates obstructive sleep apnea (OSA), and activation of the tongue musculature can lessen OSA. The hypoglossal motor nucleus (HMN) innervates the tongue muscles but there is no pharmacological agent currently able to selectively manipulate a channel (e.g., Kir2.4) that is highly restricted in its expression to cranial motor pools such as the HMN. To model the effect of manipulating such a restricted target, we introduced a “designer” receptor into the HMN and selectively modulated it with a “designer” drug. We used cre-dependent viral vectors (AAV8-hSyn-DIO-hM3Dq-mCherry) to transduce hypoglossal motoneurons of ChAT-Cre^+^ mice with hM3Dq (activating) receptors. We measured sleep and breathing in three conditions: (i) sham, (ii) after systemic administration of clozapine-N-oxide (CNO; 1 mg/kg) or (iii) vehicle. CNO activates hM3Dq receptors but is otherwise biologically inert. Systemic administration of CNO caused significant and sustained increases in tongue muscle activity in non-REM (261 ± 33% for 10 hrs) and REM sleep (217 ± 21% for 8 hrs), both P < 0.01 versus controls. Responses were specific and selective for the tongue with no effects on diaphragm or postural muscle activities, or sleep-wake states. These results support targeting a selective and restricted “druggable” target at the HMN (e.g., Kir2.4) to activate tongue motor activity during sleep.

Obstructive sleep apnea (OSA) is a common and serious breathing disorder characterized by repeated episodes of upper airway obstruction and asphyxia during sleep^1^,^2^,^3^,^4^. Treatment with continuous positive airway pressure is effective when the prescribed pressures are tolerated and present throughout the night, but sub-optimal adherence and effectiveness are commonly reported^5^; other treatment mainstays such as oral appliances or surgery can be less effective^6^. In OSA, however, it is key to note that the upper airway remains open in wakefulness and closes only in sleep. OSA is, therefore, ultimately caused by sleep-dependent changes in neuromodulators acting on critical pharyngeal motor pools, such as the hypoglossal that drives the muscles of the tongue. As a consequence, OSA is open to pharmacotherapy to counteract the effects of such sleep-dependent changes on pharyngeal muscle activity, although no such effective pharmacotherapy currently exists (for reviews see refs 7, 8, 9, 10, 11). A key desired outcome of an OSA pharmacotherapy is to sustain pharyngeal muscle activity during sleep at waking levels. If this effect can be achieved then the potential of therapeutic efficacy is realistic.

We showed that manipulation of certain potassium channels at the hypoglossal motor pool can activate the tongue musculature throughout sleep to waking levels^12^,^13^. Of note, the inward-rectifying potassium 2.4 channel (Kir2.4) is expressed almost exclusively in the cranial motor pools that modulate pharyngeal muscle tone, and not in other brain regions^14^,^15^,^16^. This localized expression makes Kir2.4 a therapeutic target of high interest, but there are no currently available small molecules to specifically modulate it^13^,^16^.

In the current absence of an agent that is able to selectively manipulate such a locally expressed channel at the motor pools critical to OSA pathogenesis we instead modelled this system by artificially introducing a “designer” receptor into this motor pool and then selectively modulated it with a “designer” drug. The present study identifies, in principle, if systemic ingestion of a drug that selectively manipulates a regionally restricted “druggable” target at the hypoglossal motor pool can sustain the activity, during sleep, of a muscle that is ultimately critical to OSA pathogenesis.

Designer Receptor’s Exclusively Activated by Designer Drugs (“DREADDs”) are bioengineered G-protein coupled receptors that are exclusively modulated by the chemical clozapine-N-oxide (CNO), which is otherwise bio-inert and blood brain barrier diffusible^17^,^18^,^19^. This technique is used to ‘tag’ discrete cell populations with the artificial receptor and then selectively modulate them via systemic drug ingestion that only affects that target^17^,^18^,^19^. Localized incorporation of DREADDs into hypoglossal motoneurons allows us to model the restricted expression of a pharmacological target in this motor pool that is highly relevant to OSA pathogenesis. Here we used a combination of genetic, pharmacological and electrophysiological approaches in mice to incorporate a viral vector carrying the chosen DREADD transgene (hM3Dq) locally into the hypoglossal motor nucleus to test three hypotheses: (i) systemic injection of CNO will activate the tongue musculature; (ii) the tongue muscle activation will be sustained throughout sleep; (iii) and that responses to CNO will be selective and specific to the tongue musculature.

This research is significant because it identifies that introducing an ‘artificial’ receptor and its small molecule activator in a murine model has beneficial effects in driving the hypoglossal motor circuit that is critical to preventing OSA in humans. The results provide proof-of-principle to develop and test small molecules acting upon *‘natural’* drug targets that are similarly locally expressed at the cranial motor pools relevant to OSA.

## Methods

### Animals

Experiments were performed on 3–6 month old, male, transgenic C57BL/6 mice (mean ± SD body weight = 25.4 ± 3.0 g, range: 19.0–29.4 g), genetically modified to express Cre-recombinase exclusively in cholinergic neurons (ChAT-Cre) (B6; 129S6-Chat^tm2(cre)Lowl^/J; The Jackson Laboratory, Bar Harbor, Maine, USA). All procedures complied with the recommendations of the Canadian Council on Animal Care, and the University of Toronto Animal Care Committee approved the protocols. Mice were exposed to a 12 hour light-dark cycle (7:00 h lights on), had access to food and water *ad libitum*, and were housed individually following surgery.

### Surgical Procedures

#### General procedures

Two sterile surgeries were performed separated by a 2-week recovery period. Both surgeries were performed under general anesthesia induced and maintained by isoflurane. Mice were first placed in an induction chamber and general anesthesia was induced by transiently passing 5% isoflurane in oxygen through the chamber at a rate of 1 L/min. Following anesthetic induction the mice were placed on a heating pad and positioned within a stereotactic frame (model 962; Kopf Instruments, Tujunga, CA, USA), and an anesthetic mask was placed over the snout. The animals spontaneously breathed 1 to 2.5% isoflurane in oxygen for the duration of the surgical procedures. Appropriate anesthetic depth was judged by abolition of the pedal withdrawal reflex. The mice additionally received subcutaneous injections of lactated ringers solution (0.3 ml) for fluid loading, ketoprofen (5 mg/kg) to reduce potential postoperative pain and received an intra-peritoneal injection of dexamethasone (6.5 mg/kg) to reduce potential brain inflammation.

#### Viral constructs injected into the hypoglossal motor nucleus

Two separate viral constructs were used for the experiments: (i) rAVV8/hSyn-DIO-hM3Dq-mCherry microinjected into the hypoglossal motor nucleus of 15 mice, and (ii) rAVV8/hSyn-DIO-mCherry (i.e., lacking hM3Dq) microinjected into the hypoglossal motor nucleus of 8 mice. A recombinant adeno-associated viral vector of serotype 8 (rAAV8) was selected because this serotype produces rapid, robust and sustained transgene expression in neurons^20^. Additionally, the use of a human synapsin 1 (hSyn) promoter confines viral transduction to neuronal cells, while linking the receptor transgene to the mCherry construct allows visualization of effective receptor expression by fluorescent microscopy. Finally, using a viral construct where the receptor transgene is contained within a double-floxed inverse open (DIO) reading frame confers transgene expression that is dependent on Cre-recombinase. Since motoneurons are (by definition) cholinergic, and ChAT-Cre mice express Cre-recombinase exclusively in cholinergic neurons, the hypoglossal motoneurons of ChAT-Cre mice express the Cre-recombinase enzyme necessary for the mCherry-tagged transgene to be transduced following local microinjection into the hypoglossal motor nucleus.

A syringe pump (#704504, Harvard Apparatus, Holliston, MA, USA) was used to microinject the viral constructs in a solution containing 350 mM NaCl and 5% D-Sorbitol in phosphate buffered saline (University of North Carolina (UNC) Vector Core, Chapel Hill, NC, USA). This injection was performed using a 50 μl Hamilton syringe (Model 750 N SYR; Hamilton Company, Reno, NV, USA) connected to a 28gauge stainless steel cannula (Plastics One; Roanoke, VA, USA) via polyethylene tubing (PE No.20; VWR, Radnor, PA, USA). The syringe and tubing were filled with mineral oil and the cannula-end of the tubing contained the viral vector. A pocket of air separated the two liquids.

The hypoglossal motor nucleus was targeted using stereotaxic coordinates as determined from preliminary experiments in this mouse strain. Inserting the cannula at the midline (i.e., at a medial-lateral coordinate of 0 mm) targeted both hypoglossal motor nuclei at a mean ( ± SEM) anterior-posterior coordinate of −6.80 ± 0.08 mm, range: −6.39 to −7.41 mm from bregma). In practice the posterior coordinate in each mouse was indicated by the intersection of the parietal and inter-parietal bones; this intersection of bone sutures at the midline was used as it marked the posterior edge of the skull from which to access the hypoglossal motor nuclei. The cannula was then lowered through the skull to a dorsal-ventral coordinate of −6.0 mm from bregma and a 200 nl viral injection was delivered at a rate of 50 nl/min. After delivery of the viral bolus, six minutes were allowed to pass before the cannula was slowly withdrawn. This time allowed the solution containing the virus constructs time to diffuse, preventing it from being drawn back up the tract created by the cannula upon withdrawal. The skin was then rehydrated with saline after the cannula was removed and the incision was closed with dissolvable sutures (Polysorb, Lot #B2D0946X; Covidien, Dublin, Ireland). The sutures were then cleaned with alternating swabs of povidone-iodine, and a 70% ethanol solution and topical antibiotic ointment was also applied (Polysporin; Johnson & Johnson Inc., New Brunswick, NJ, USA).

Mice recovered in a clean cage warmed by a heating pad for 24 hours, and were provided soft food and hydration gel for the first day after surgery. The animals were judged to have recovered fully when they resumed normal locomotor activity, grooming, drinking, and eating behaviour. Mice recovered for two weeks before the second surgery that involved implantation of electrodes for the chronic recording of sleep and breathing.

#### Instrumentation for chronic recordings of sleep and breathing

Mice were re-anesthetized two weeks after the first procedure using the methods described above (see section: *General procedures*). The mice were then implanted with electroencephalogram (EEG) and neck electromyogram (EMG) electrodes for the determination of sleep–wake states, and with tongue and diaphragm electrodes for EMG recordings^21^,^22^,^23^. For each mouse, an electrode plug was custom made before surgery containing the EEG and EMG electrodes. The three skull electrodes (i.e., bipolar EEG electrodes plus a common reference) consisted of insulated stainless steel wire (AS631; Cooner Wire, Chatsworth, CA, USA) attached to a stainless steel screw (No. 0090 × 3/32; HRS Scientific, Anjou, QC, Canada). The neck and diaphragm EMG electrodes were made with the same wire but the ends were knotted loops. The tongue EMG electrodes were made from a more rigid and lower gauge stainless steel wire (AS636; Cooner Wire, Chatsworth, CA, USA) with a knotted end of ~1 mm diameter. Each electrode was soldered onto individual pins on a male plug (ED90267-ND; Digi-Key, Thief River Falls, MN, USA) that contained the four pairs of electrode leads and the common reference.

For recording muscle activity, the pairs of the insulated stainless-steel wires were implanted subcutaneously into the dorsal neck and tongue muscles. These muscles were exposed by blunt dissection after small midline incisions over their respective regions (dorsum of neck and submentum). The flexible wire electrodes were also placed onto the diaphragm via an abdominal incision. All wires were tunneled subcutaneously from a cranial incision and the other incisions were then closed using absorbable sutures and covered with antibiotic ointment. The mice were then placed in a stereotaxic apparatus and the small stainless steel screws attached to the EEG electrodes were attached to the skull along with an anchor screw. Dental cement was used to affix the electrode plug onto the skull. The remaining cranial incision was then closed around the dental cement that contained the electrode plug using absorbable sutures and covered with antibiotic ointment.

### Experimental Protocol

#### Habituation

Two weeks after the instrumentation for chronic recordings of sleep and breathing, and two days prior to experimentation, the mice were habituated to the recording environment. The recording environment consisted of the mouse housing chamber fitted with a custom made Plexiglas top that allowed for passage of the recording cable. At the start of the habituation period the electrode plug of each mouse was connected to the female plug attached to the flexible recording cable (CW7117; Cooner Wire, Chatsworth, CA) that allowed free movement within the chamber.

#### Protocol and sequence of interventions

After two days habituation the experimental recordings took place over 72 hours and consisted of three, 22-hour recording periods. The first recording period (sham) was used to collect data from each animal under conditions in which there was no intervention and the animals were uninterrupted and behaved freely. On the second and third days, the mouse received an intra-peritoneal injection of either clozapine-N-oxide (CNO, 1 mg/kg in a vehicle consisting of 0.05% dimethyl sulfoxide in 0.9% saline), or they received the vehicle alone. The order of CNO or vehicle alternated between consecutive mice. The recordings ran from 07:00 hrs to 05:00 hrs the following day, and interventions (sham, CNO or vehicle) occurred at 09:00 hrs. Mice were video recorded for the duration of the testing period and monitored remotely to avoid disturbances.

#### Recordings

The EEG and EMG signals were amplified and filtered (Model 1700; A-M Systems, Sequim, WA, USA). The EEG was filtered between 1 and 1000 Hz, and the EMG signals between 100 Hz and 1000 Hz. In addition to the raw EMG signals, the moving time averages were also obtained from the neck, tongue and diaphragm using time constants of 50 ms, 50 ms and 100 ms respectively (Model MA-821, CWE Inc., Ardmore, PA, USA). The electrocardiogram was removed from the diaphragm using an electronic blanker (Model SB-1, CWE Inc., Ardmore, PA, USA). Signals were digitized and recorded on a computer (Spike 2 software, 1401 interface, CED, Cambridge, UK). The raw EMG and EEG signals were sampled at 2000 Hz, and the moving-time averaged signals were sampled at 1000 Hz.

### Data Analysis

#### Identification of sleep-wake states

Experimental recordings consisted of multiple two hour data files analyzed in consecutive 5 sec time bins. The moving time averaged signals for the neck, tongue and diaphragm signals were analyzed during periods identified as active wakefulness, quiet wakefulness, non-rapid eye movement (non-REM) and REM sleep. Active wakefulness was identified as periods characterized by high frequency and low amplitude EEG activity, accompanied by high levels of neck EMG activity associated with overt behaviors such as eating, grooming, drinking and locomotion. Quiet wakefulness was identified as periods also containing high frequency and low amplitude EEG activity, but with lower levels of neck muscle activity than active waking (the thresholds used to separate periods of active and quiet wakefulness are identified below). Non-REM sleep was identified as periods characterized by low frequency and high amplitude EEG activity, coupled with low tonic EMG activity. REM sleep was identified as periods containing higher frequency and low amplitude EEG activity, but minimal neck muscle activity.

#### EMG data

The algorithm used to separate periods of active and quiet wakefulness from the neck EMG signal in each mouse was generated using MATLAB^®^ (The Mathworks, Inc. 2013). A state of active wakefulness was assigned if either of two conditions were met: (i) the mean neck EMG activity in a 5 sec epoch was greater than 4% of the single maximum neck activity recorded in the dataset for that mouse, or (ii) if more than 10% of the EMG data in the 5 sec epoch exceeded 8% of this maximum value. The algorithm was generated and validated by measuring the EMG and EEG data and comparing with the video recordings of animal behavior scored for periods of active and quiet wakefulness. In addition, the algorithm was further validated in two experimental animals under each of the three experimental conditions (i.e. sham, CNO and vehicle) for 6 hrs each, i.e., 36 hours of data in total. Visual and automated scoring of active and quiet wakefulness differed by 3.0 ± 0.9% (SEM), i.e., were the same an average of 97% of the time.

The tongue EMG signal was averaged within each 5 sec epoch. Tongue muscle activity was not separated into respiratory and tonic components, as tonic activity was almost always present except for transient non-respiratory activations associated with behaviors such as drinking, grooming and eating. The peaks and troughs of the moving time averaged diaphragm signal were identified using a custom-made script, and values of average diaphragm amplitude and respiratory rates were determined for each 5 sec epoch^21^,^23^. Respiratory rates and diaphragm amplitudes were only analyzed in periods of quiet waking, non-REM and REM sleep due to artefacts associated with non-respiratory activities present during behaviors in active wakefulness.

Values of neck and tongue muscle activities, diaphragm amplitude and respiratory rate for each epoch were averaged to produce a grand mean for each variable, in each sleep-wake state, in each experimental condition (sham, CNO or vehicle), for each mouse.

### Histology

At the end of the experiments the mice were overdosed with an intra-peritoneal injection of tribromoethanol (0.025 ml/g) and perfused with 0.1 M phosphate buffered saline (PBS), followed by 4% paraformaldehyde in PBS. The brains of all mice and the tongues of seven mice were removed and fixed in paraformaldehyde overnight and then cryo-protected in 30% sucrose in PBS. Prior to sectioning, the brains and tongues were placed on dry ice for 30 min and then stored at −20 °C for 1 hr. The medulla was cut into 30 μm coronal sections and the tongue into 50 μm sagittal sections using a cryostat (Leica CM1850; Wetzlar, Hesse, Germany). The cell nuclei in brain slices were stained using 4′,6-Diamidine-2′-phenylindole dihydrochloride (DAPI, Vector Laboratories; Burlington, ON, Canada), with the sections mounted on glass slides and sealed with Fluoromount (Sigma-Aldrich; Oakville, ON, Canada). Expression and localization of the DREADDs transgenes in the medulla was confirmed by visualizing the mCherry protein expression using an Axioscan Z1 slide scanner (Texas Red filter) connected to a computer running Zen 2 blue edition (Carl Zeiss Microscopy; North York, ON, Canada). The extent of the mCherry expression was traced for each mouse. The mouse brain atlas^24^ was used for reference. The location of the electrode tracts in the tongue muscle was identified histologically using the sagittal sections of tongue tissue. Those sections were mounted, stained with Neutral Red (72210-100G; Sigma-Aldrich, Oakville, ON, Canada), and visualized using a slide scanner (brightfield).

### Statistical Analysis

Each mouse served as its own control as all interventions were performed in a single experiment, allowing for comparisons across experimental conditions (i.e. sham, CNO and vehicle) and sleep-wake states. The analyses performed for each statistical test are detailed in the text where appropriate. For the two-way analyses of variance with repeated measures (ANOVA-RM), the two factors were experimental condition (i.e., sham, CNO and vehicle) and sleep-wake state (i.e., active wakefulness, quiet wakefulness, non-REM sleep and REM sleep). For all comparisons, differences were considered significant if the null hypothesis was rejected at P < 0.05 using a two-tailed test. Where post hoc comparisons were performed after ANOVA-RM, Bonferroni-corrected P value was used to test statistical significance. All analyses were performed using Sigmastat (SPSS, Chicago, IL). Values are shown as means ± the standard error of the mean (SEM) unless otherwise indicated as standard deviation (SD).

## Results

### Expression of the DREADDs transgene at the hypoglossal motor pool

**Figure 1:**
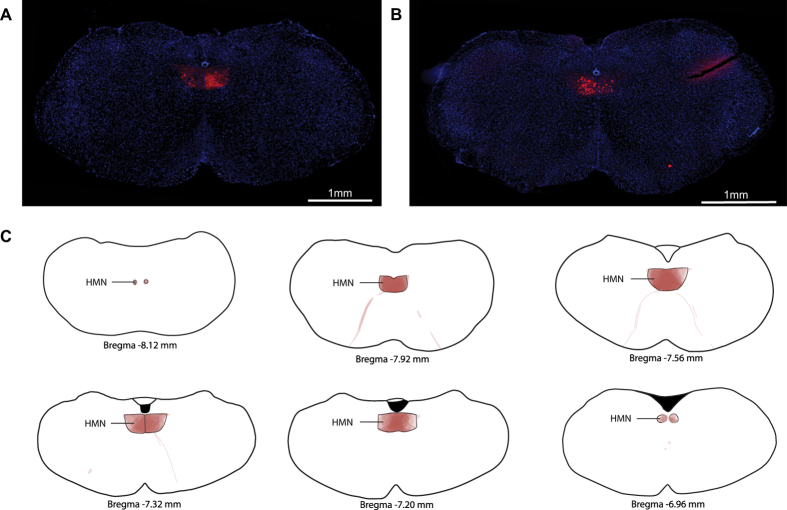
Expression of the DREADDs transgene at the hypoglossal motor pool. Example fluorescent microscopy images illustrating the effective transduction of the viral vector, as indicated by mCherry expression (red) at the hypoglossal motor nucleus (HMN) in coronal brain sections from two mice: (**A**) viral vector containing the DREADD transgene (rAVV8/hSyn-DIO-hM3Dq-mCherry), and (**B**) viral vector lacking the DREADD transgene (rAVV8/hSyn-DIO-mCherry, i.e., lacking hM3Dq). The group data (**C**) show the regions of mCherry expression traced from each section in each mouse (n = 11), with these regions then superimposed on the standard reference brain sections. A high degree of overlap of the regions of mCherry expression across the different mice is indicated by the intensity of the red colour from the superimposed traces, whereas the lighter shading indicates lesser overlap of these regions between mice. These data show effective mCherry expression in the hypoglossal motor nucleus. That the expression is effectively targeted to hypoglossal motoneurons is also indicated by the mCherry expression in the tracts of the hypoglossal motor nerves arising from this motor pool (**C**). The cell nuclei in the brain slices (**A**,**B**) were stained using 4′,6-Diamidine-2′-phenylindole dihydrochloride (DAPI).

Figure 1A shows an example from one of the 8 mice in which the hypoglossal motor pool was injected with the viral vector incorporating the DREADDs transgene. This example shows effective transduction as indicated by mCherry expression (Fig. 1A). Likewise, Fig. 1B shows an example from one of the 3 mice in which the hypoglossal motor pool was injected with the viral vector that lacked the DREADDs transgene (i.e., the inactive virus control). This example also shows transduction of the inactive virus as indicated by mCherry expression (Fig. 1B). These single examples as well as the group data (Fig. 1C) show that transduction was largely confined to the boundaries of the hypoglossal motor nucleus across animals.

A total of 345 hours (i.e., 248,400 5-sec epochs) of physiologic data and video recordings were collected and analyzed from these 11 mice (i.e., 8 with the viral vector incorporating the DREADDs transgene at the hypoglossal motor pool, and 3 inactive virus controls). The 345 hours comprised up to 33 hours of recordings per mouse, encompassing 11 hours for each of the sham and vehicle conditions (i.e., the two control periods) and the CNO condition (i.e., the period of the test intervention). This 11 hours of analyzed data per condition in each mouse consisted of 2 hours baseline recordings pre-intervention followed by 10 hours post-intervention, but with the first hour not analyzed to minimize any potential confound related to an acute behavioral activation result from handling and the intra-peritoneal injection. In 3 of the 8 mice transduced with the viral vector incorporating the DREADDs transgene, 27 hours of physiologic data and video recordings were collected (i.e., not the full 33 hours). This difference was because data were not collected during the 2 hours baseline recordings pre-intervention in these 3 mice, such that 9 hours of data was collected for each of the three experimental conditions in these animals.

### Tongue muscle activity across sleep-wake states

**Figure 2:**
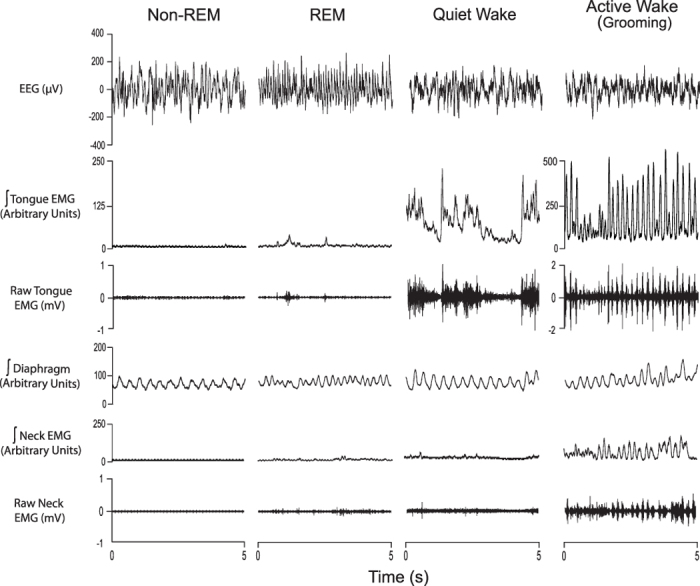
Tongue muscle activity across sleep-wake states. Examples from one mouse showing the electroencephalogram (EEG) and the raw and/or integrated electromyograms (EMGs) of the tongue, neck and diaphragm muscles during non-REM and REM sleep, as well as during quiet and active wakefulness. The hypoglossal motor nucleus of this mouse was injected with the viral vector containing the DREADD transgene (rAVV8/hSyn-DIO-hM3Dq-mCherry) and the sample recordings are from the sham condition. Note the phasic tongue muscle activations during active wakefulness (grooming) but the lack of respiratory-related activity across sleep-wake states.

Stereotypical motor acts such as eating and grooming were associated with phasic activation of the tongue musculature in these freely behaving mice (Fig. 2). Periods of quiet wakefulness were mainly associated with tonic muscle activity interspersed with occasional phasic activations, but respiratory-related activity was not observed (Fig. 2). Tongue muscle activity was minimal in both non-REM and REM sleep, except for periods of transient tongue muscle activations during REM sleep that also occurred in the neck muscle (Fig. 2).

**Figure 3:**
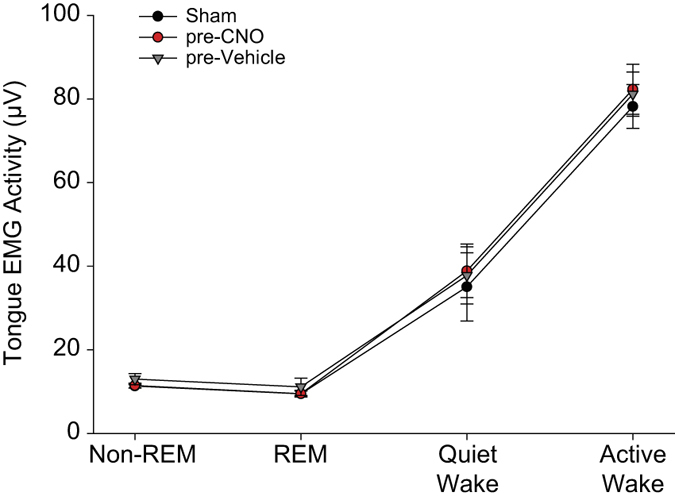
Group data showing tongue muscle activity varies across sleep-wake state pre-intervention. Tongue muscle activities recorded in the 2 hours of baseline recordings (i.e., *before* any interventions) were indistinguishable between the three experimental conditions (i.e., sham, pre-vehicle and pre-CNO). Tongue muscle activity varied across sleep-wake states, being higher in active wakefulness compared to quiet wakefulness, and higher in quiet wakefulness compared to sleep. Values are mean ± SEM.

The group data showed that tongue muscle activity recorded in the 2 hours of baseline recordings (i.e., *before* any interventions) were indistinguishable between the three experimental conditions (i.e., sham, before vehicle and before CNO, F_2,14_ = 1.34, P = 0.292, two-way ANOVA-RM). However, tongue muscle activity did vary significantly across sleep-wake states (F_3,21_ = 64.63, P < 0.001, two-way ANOVA-RM, Fig. 3). Further analyses confirmed that tongue muscle activity was higher in active wakefulness compared to quiet wakefulness (t_7_ = 7.68, P < 0.001, post-hoc paired t-test), higher in quiet wakefulness compared to non-REM sleep (t_7_ = 4.49, P = 0.001), but of similar magnitude between non-REM and REM sleep (t_7_ = 0.25, P = 1.00).

**Figure 4:**
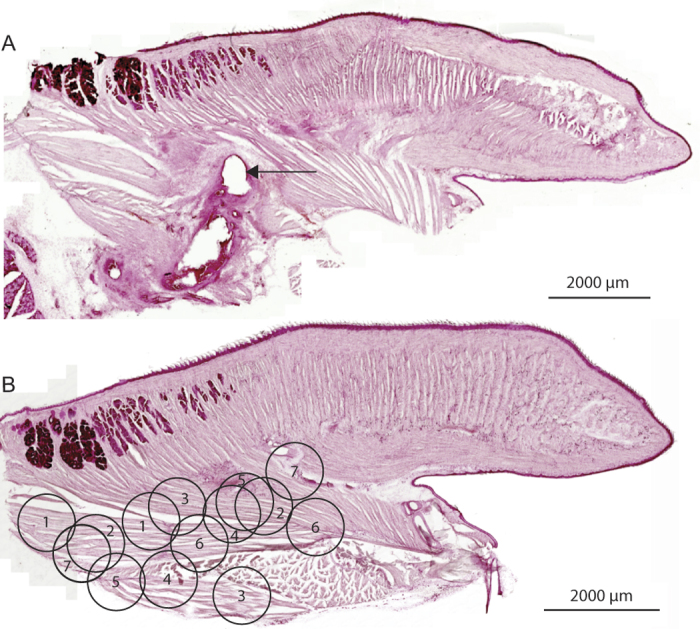
Location of tongue muscle electrodes. The arrow on the sagittal section of the tongue shows the lesion left by the distal tip of one of the recording electrodes from one mouse (**A**). The locations of the distal tips of both electrodes are also shown for each of the seven mice analyzed (each mouse is indicated by a different number), with the placements superimposed on a reference sagittal section of the tongue (**B**). The electrodes were located in the tongue musculature.

The tongues of seven mice were analyzed post-mortem to confirm the location of the electrode tips in the tongue musculature. Figure 4A shows an example of the lesion left by one electrode in the tongue of one mouse, and Fig. 4B shows the representations of the locations of the bipolar electrodes in the seven mice.

### Tongue muscle activation in response to systemic injection of CNO in mice with hM3Dq receptors transduced at the hypoglossal motor pool

**Figure 5:**
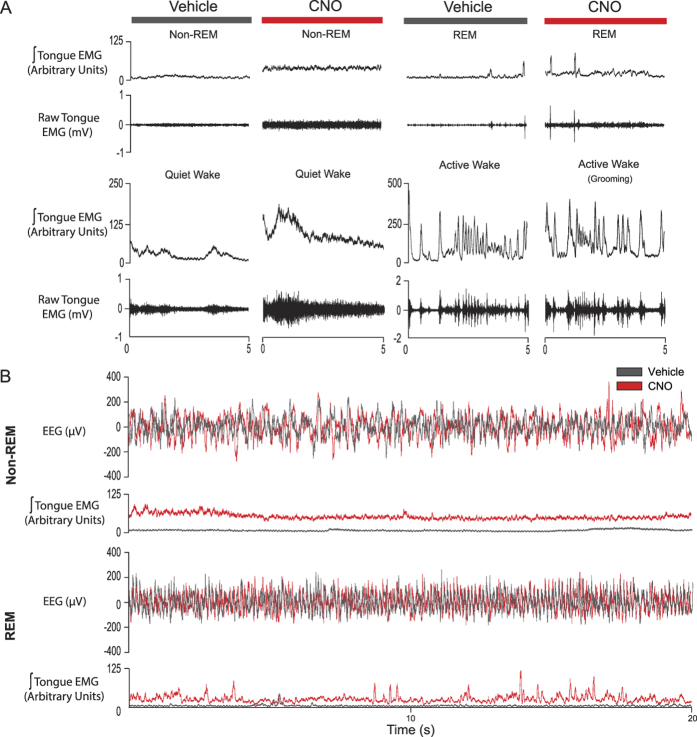
Tongue muscle activation in response to systemic injection of CNO in mice with hM3Dq receptors transduced at the hypoglossal motor pool. Examples of tongue muscle activation in response to systemic injection of CNO in one mouse with the viral vector containing the DREADD transgene (rAVV8/hSyn-DIO-hM3Dq-mCherry) at the hypoglossal motor pool. Note the clear tonic tongue muscle activation after CNO compared to vehicle that is present in non-REM and REM sleep, and quiet wakefulness (**A**). The increase in active wakefulness after CNO is partially masked by ongoing behaviors. Longer periods of non-REM and REM sleep are also shown (**B**) to illustrate that the increased tongue muscle activity elicited by CNO was also sustained throughout the sleeping periods. In the lower traces the tongue muscle activity after vehicle (black) and CNO (red) are superimposed on the same scale to illustrate the increase after CNO. The EEG traces are also superimposed for the vehicle (black) and CNO (red) conditions. Abbreviations are as for Fig. 2.

Figure 5A shows an example of tongue muscle activity recorded after systemic injection of CNO in a mouse with hM3Dq receptors transduced at the hypoglossal motor pool, compared to the same mouse after injection of vehicle alone. Note the tonic activation of the tongue muscle that is clearly observed in both non-REM sleep, REM sleep and quiet waking after CNO compared to the vehicle control (Fig. 5A). No respiratory-related activation was elicited. Also note that the increased tongue muscle activity that is normally present in active wakefulness partially masks this excitatory effect of CNO (Fig. 5A). The increased tongue muscle activity elicited by CNO was also sustained, as shown by the examples including longer periods of non-REM and REM sleep in Fig. 5B.

**Figure 6:**
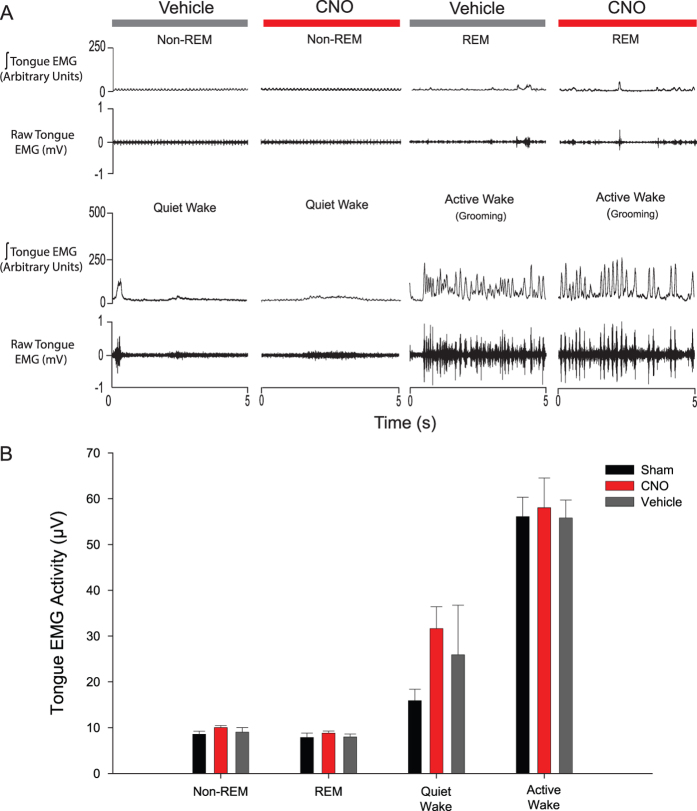
Lack of effect of CNO in the mice in which the hypoglossal motor pool was transduced with the viral vector that lacked the DREADDs (hM3Dq) transgene, i.e., the inactive virus controls. Example (**A**) and group data (**B**) showing the *lack* of tongue muscle activation in response to systemic injection of CNO in a mouse with the *inactive* viral vector expressed at the hypoglossal motor pool. Values are mean + SEM. Abbreviations as for Fig. 2.

That the excitatory effect of CNO on tongue muscle activity is mediated by its effect on hM3Dq receptors at the hypoglossal motor pool is shown by the *lack of effect* of CNO in the mice in which the hypoglossal motor pool was transduced with the viral vector that *lacked* the DREADDs (hM3Dq) transgene, i.e., the inactive virus controls (Fig. 6).

**Figure 7:**
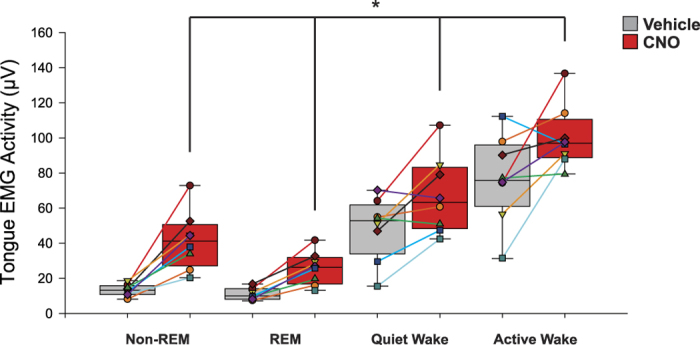
Individual and average excitatory effects of CNO on tongue muscle activity across sleep-wake states. Box and whisker plots showing the individual and group (i.e., mean, 25^th^ and 75^th^ percentiles, maximum and minimum) tongue muscle activities recorded over four hours after systemic injection of CNO or vehicle in the mice with hM3Dq receptors transduced at the hypoglossal motor pool. Statistical analysis showed that tongue muscle activity was significantly higher after systemic injection of CNO compared to vehicle, and this excitatory effect of CNO did not depend on the prevailing sleep-wake state (indicated by the symbol *). There was also a significant effect of sleep-wake state on tongue muscle activity, being higher in active wakefulness compared to quiet wakefulness, and higher in quiet wakefulness compared to sleep. See text for further details.

Figure 7 shows the individual and average excitatory effects of CNO on tongue muscle activity across sleep-wake states compared to the vehicle controls in the first four hours after injection. Statistical analysis showed that there was a significant effect of experimental condition on tongue muscle activity (F_2,14_ = 20.53, P < 0.001, two-way ANOVA-RM, Fig. 7). Further analysis identified that tongue muscle activity was significantly higher after systemic injection of CNO compared to both the vehicle and sham controls (both t_7_ > 4.43, P < 0.003, post-hoc paired t-test), and that tongue muscle activity in the vehicle and sham conditions were of similar magnitude (t_7_ = 0.40, P = 1.00). There was also a significant effect of sleep-wake state on tongue muscle activity (F_3,21_ = 81.26, P < 0.001), with tongue activity being higher in active wakefulness compared to quiet wakefulness (t_7_ = 6.42, P < 0.001, post-hoc paired t-test), higher in quiet wakefulness compared to non-REM sleep (t_7_ = 6.22, P < 0.001), but of similar magnitude between non-REM and REM sleep (t_7_ = 1.39, P = 1.00). Also importantly, the effect of experimental condition on tongue muscle activity was not dependent on the prevailing sleep-wake state (F_6,41_ = 1.11, P = 0.376), showing that sleep-wake state exerted state-dependent modulation of the hypoglossal motor pool and tongue muscle activity even in the presence of the significant motor activation elicited by CNO.

Overall, the data in Fig. 7 showed that systemic administration of CNO increased tongue muscle activity in non-REM and REM sleep by 312 ± 33% and 250 ± 20% respectively in the first four hours after injection compared to the vehicle controls. The levels of tongue muscle activity achieved after CNO in non-REM and REM sleep reached 93 ± 12% and 58 ± 8% of those measured during quiet wakefulness after the vehicle control in the same time period.

### Responses to systemic injection of CNO were specific to the tongue musculature

The responses elicited by systemic injection of CNO (Figs 5 and 7) were specific to the tongue as there we no changes in other physiological variables.

#### Respiratory rate

**Figure 8:**
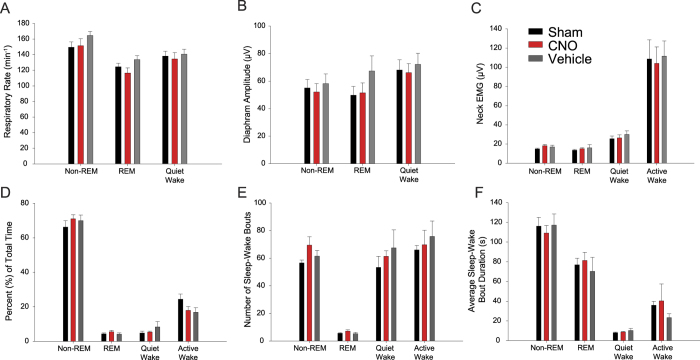
Responses to systemic injection of CNO were specific to the tongue musculature as there were no effects on other physiological variables. There were no significant differences between the three experimental conditions (i.e., sham, and responses to vehicle or CNO) on respiratory rate (**A**), diaphragm amplitude (**B**), neck muscle activity (**C**), the percent time spent in each sleep-wake state (**D**), the number of sleep-wake bouts (**E**) or their duration (**F**). Values are mean + SEM.

There was no effect of experimental condition (i.e., sham, vehicle or CNO) on respiratory rate (F_2,14_ = 2.22, P = 0.142, two-way ANOVA-RM, Fig. 8A). There was a significant effect of sleep-wake state on respiratory rate (F_2,14_ = 15.30, P < 0.001), with rate being lower in REM sleep than non-REM sleep (t_7_ = 5.36, P < 0.001, post-hoc paired t-test). The effect of sleep-wake state on respiratory rate did not depend on experimental condition (F_4,20_ = 0.39, P = 0.814). Respiratory rates were only analyzed in periods of quiet waking, non-REM and REM sleep due to artefacts and non-respiratory activity present during behaviors in active waking.

#### Diaphragm amplitude

There was no significant effect of experimental condition (sham, vehicle or CNO) on diaphragm amplitude (F_2,14_ = 2.85, P = 0.091, two-way ANOVA-RM, Fig. 8B). There was also no significant effect of sleep-wake state on diaphragm amplitude (F_2,14_ = 3.06, P = 0.079), and no effect of experimental condition that depended on sleep-wake state (F_4,28_ = 0.39, P = 0.814). As for respiratory rate, diaphragm amplitudes were only analyzed in periods of quiet waking, non-REM and REM sleep due to artefacts and non-respiratory activity present during behaviors in active wakefulness.

#### Neck muscle activity

There was no significant effect of experimental condition (sham, vehicle or CNO) on neck muscle activity (F_2,14_ = 2.69, P = 0.103, two-way ANOVA-RM, Fig. 8C). There was a significant effect of sleep-wake state on neck muscle activity (F_3,21_ = 10.48, P < 0.001) that did not depend on experimental condition (F_6,42_ = 1.07, P = 0.398). Further analyses showed that neck muscle activity was higher in active wakefulness compared to quiet wakefulness and sleep (all t_7_ > 4.33, all P < 0.003, post-hoc paired t-tests, Fig. 8C).

#### Sleep-wake architecture

There was no effect of experimental condition (sham, vehicle or CNO) on the percent time spent in each sleep-wake state, the number of sleep-wake episodes, or their duration (all F_2,14_ < 1.05, P > 0.378, two-way ANOVA-RM, Fig. 8D–F).

### Persistent tongue muscle activation in response to systemic injection of CNO

**Figure 9:**
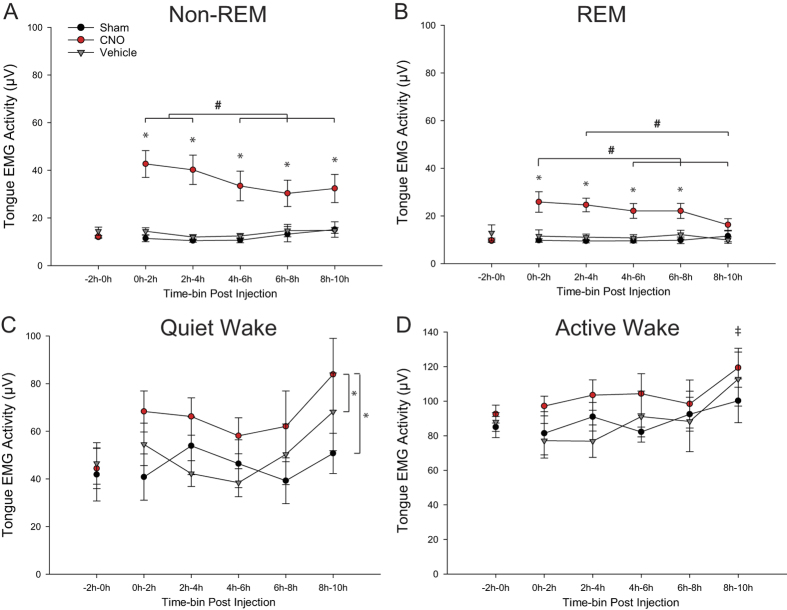
Persistent tongue muscle activation in response to systemic injection of CNO. Group data showing significant and sustained tongue muscle activation across sleep-wake states after systemic injection of CNO in the mice with hM3Dq receptors transduced at the hypoglossal motor pool. Data are shown in the baseline period pre-intervention and up to 10 hours post-intervention for each of the CNO, vehicle and sham conditions. For each of non-REM and REM sleep and quiet wakefulness (**A**–**C**), there was a significant effect of experimental condition on tongue muscle activity, with activity after CNO being significantly increased compared to both the vehicle and sham conditions. This effect of experimental condition just failed to reach statistical significance in active wakefulness (**D**, P = 0.052). The symbol ‘*’ indicates significant increases in tongue muscle activity after CNO, compared to the same time-bins in the vehicle and sham conditions from post-hoc analyses. The symbol ‘#’ indicates significant changes in tongue muscle activity over time after CNO (i.e., between time-bins) from post-hoc analyses. The symbol ‘‡’ in panel D indicates that tongue muscle activity during the 8^th^ to 10^th^ hour of recording post-intervention in active wakefulness (i.e., 17:00 to 19:00 hrs clock time) was significantly increased compared to earlier time points, independent of experimental condition. See text for further details.

Tongue muscle activity was analyzed up to 10 hours post-intervention in each of the CNO, vehicle and sham conditions and these data are shown in Fig. 9. Although there was significant tongue muscle activation that persisted for hours after CNO (Fig. 9, and analysed below), it is important to note that activity returned to similar baseline values each day before the interventions as measured in each sleep-wake state for each experimental condition, i.e., sham, vehicle or CNO (each F_2,8_ < 2.68, P > 0.128, one-way ANOVA-RM, Fig. 9).

#### Non-REM sleep

Figure 9A shows that in non-REM sleep there was a significant effect of experimental condition on tongue muscle activity (F_2,14_ = 18.19, P < 0.001, two-way ANOVA-RM) that depended on time post-intervention (F_8,56_ = 5.77, P < 0.001). Post-hoc analyses showed that tongue muscle activity after CNO was significantly increased compared to both the vehicle and sham conditions for each of the time bins analyzed up to 10 hours post-intervention (all t_7_ > 3.60, P < 0.01, paired t-tests). Tongue muscle activity during non-REM sleep did not change over time in the sham and vehicle conditions (all t_7_ < 2.25, P > 0.276, post-hoc paired t-tests), but did change over the 10 hour measurement period after CNO injection; activity in the first four hours post-CNO was statistically greater than during hours four to ten post-CNO (each t_7_ > 3.24, P < 0.02, post-hoc paired t-tests, Fig. 9A). Overall, systemic administration of CNO increased tongue muscle activity in non-REM by 261 ± 33% over this 10 hr time period post-injection compared to the vehicle controls.

#### REM sleep

There was also a significant effect of experimental condition on tongue muscle activity in REM sleep (F_2,14_ = 28.35, P < 0.001, two-way ANOVA-RM) that depended on time post-intervention (F_8,40_ = 4.78, P < 0.001). Tongue muscle activity after CNO was significantly increased compared to both the vehicle and sham conditions for each of the time bins analyzed up to 8 hours post-intervention (all t_7_ > 4.40, P < 0.01, post-hoc paired t-tests, Fig. 9B) but not for the 8–10 hour time bin (each t_7_ < 2.19, P > 0.118, paired t-tests). Tongue muscle activity during REM sleep did not change over time in the sham and vehicle conditions (all t_7_ < 2.07, P > 0.423, post-hoc paired t-tests), but did change over the measurement period after CNO injection; activity in the first two hours post-CNO was statistically greater than during hours four to ten post-CNO (each t_7_ > 4.24, P < 0.001, post-hoc paired t-tests, Fig. 9B), and activity in the 2–4 hr time bin post-CNO was greater than in the 8–10 hr time post-CNO (t_7_ = 4.22, P < 0.001, post-hoc paired t-test, Fig. 9B). Overall, systemic administration of CNO increased tongue muscle activity in REM sleep by 217 ± 21% over the period post-injection compared to the vehicle controls.

#### Quiet wakefulness

There was a significant effect of experimental condition on tongue muscle activity in quiet waking (F_2,14_ = 15.57, P < 0.001, two-way ANOVA-RM), with activity after CNO being significantly increased compared to both the vehicle and sham conditions (each t_7_ > 4.18, P < 0.004, post-hoc paired t-tests, Fig. 9C). The effect of experimental condition on tongue muscle activity did not depend on time post-intervention (F_8,56_ = 1.25, P = 0.289).

#### Active wakefulness

The effect of CNO on tongue muscle activity was less obvious in active wakefulness because it occurred at the same time as the engagement of the tongue muscle by behaviours such as eating, drinking and grooming. The group data in Fig. 9D showed that the effect of experimental condition on tongue muscle activity just failed to achieve statistical significance in active waking (F_2,14_ = 3.69, P = 0.052, two-way ANOVA-RM). There was an effect of time post-intervention on tongue muscle activity (F_4,28_ = 5.96, P = 0.001) that was independent of experimental condition (F_8,56_ = 0.62, P = 0.760, two-way ANOVA-RM). Further analysis showed that tongue muscle activity during the 8^th^ to 10^th^ hour of recording post-intervention (i.e., 17:00 to 19:00 hrs clock time) was significantly increased compared to earlier time points (each t_7_ > 3.15, P < 0.039, post-hoc paired t-tests, Fig. 9D), likely reflecting the enhanced motor activity in waking periods just prior to the normal active phase at lights off (19:00 hrs).

## Discussion

Here we show, using the DREADDs technology, that *systemic* administration of a compound that is otherwise biologically inert, but able to selectively activate a receptor whose expression is restricted to the hypoglossal motor pool, results in robust, selective and sustained reactivation of tongue muscle tone throughout sleep. This result obtained in a murine model establishes proof-of-principle for developing strategies to modulate a selective and restricted “druggable” target that is similarly, but naturally, present at the hypoglossal and other cranial motor pools significant to maintaining upper airway stability. One such naturally occurring and appropriately localized target is the inward-rectifying Kir2.4 channel^14^,^15^,^16^, for which there are no specific drugs in the public domain and, to our knowledge, nor have they been developed yet. We have shown previously that modulation of the generic Kir channel class with agents applied *locally* at the hypoglossal motor pool can re-activate tongue motor activity throughout sleep to normal waking levels^12^,^13^,^16^.

Development of a pharmacological strategy to maintain pharyngeal muscle tone in sleep may help treat OSA directly, or indirectly by improving upper airway stability and potentially improving effectiveness of, and adherence to, other treatments (e.g., by reducing the absolute pressure required for effective continuous positive airway pressure therapy). Although this study uses DREADDs to model the restricted expression of a pharmacological target at the hypoglossal motor pool because it is relevant to OSA pathogenesis, this study does not promote the notion of virus injection for OSA patients. We note, however, that Saper and Scammell^25^ have identified that the next generation of therapeutics in sleep medicine will be influenced by novel technologies, and that the use of viral vectors targeting specific neuronal populations to influence human brain function is being tested^26^. Moreover, in the context of the present study, the use and validation of the DREADDs technology at this model motor pool–muscle pathway is more broadly applicable to other disorders of motor function such as in spinal cord injury, motor neuron diseases and dysphagia.

### Modelling OSA pharmacotherapy

The challenges to overcome with a potential pharmacotherapy for OSA are significant. They involve identifying a strategy to selectively target the critical mechanism involved in the pathogenesis of the upper airway closure at the desired times (i.e., for hours throughout the sleeping period) with minimal off-target effects. Indeed, there are several reviews summarizing attempted pharmacotherapy for OSA^7^,^8^,^9^,^10^,^11^. Overall there has been a lack of effective and reliable clinical interventions to date^7^,^8^,^9^,^10^,^11^. However, knowledge of the basic mechanisms underlying upper airway motor suppression in sleep^12^,^13^,^16^,^27^ have led to more recent success in preventing the normal reduction of genioglossus activity from wakefulness to non-REM sleep and reducing upper airway collapsibility in human subjects^28^.

Ultimately, the key problems to be resolved for a successful pharmacotherapy are: (a) identifying an ion channel or receptor as a rational and viable pharmacological target; (b) identifying an agent that can access the desired target to exert its beneficial effect (a drug delivery problem); (c) the potential for the agent to act at other sites to obscure or oppose the beneficial response (a specificity problem); (d) any potential efficacy being obscured by unwanted side effects (a concentration-dependent, receptor-targeting and/or sensitivity problem); (e) different responses occurring in REM vs. non-REM sleep (a neurobiology problem where the neural mechanisms to be overcome at the cranial motor pools are different between non-REM and REM sleep, or the site and/or length of the upper airway obstruction may be different and/or longer in REM sleep vs. non-REM)^7^. The ultimate aim of the present study was to identify a strategy and test a simple proof-of-principle concept to overcome each of the aforementioned challenges in a murine model. Accordingly, we used a “designer” receptor inserted into a motor pool critical to OSA pathogenesis in humans to model the presence of a naturally existing target (i.e., overcoming problem “a”). We then used a “designer” drug as the agent to selectively modulate that target with no measured effects on other variables (i.e., overcoming problems “b” through “d). We subsequently showed that the agent produced sustained tongue muscle activation throughout the sleeping period (i.e., overcoming problem “e”).

Although the systemically applied CNO produced sustained (8–10 hours) and specific tongue muscle activation throughout non-REM and REM sleep (Figs 7, 8 and 9), it was also noted that the activation produced in non-REM sleep was larger than that observed in REM sleep. For example, systemic administration of CNO increased tongue muscle activity in non-REM and REM sleep by 312 ± 33% and 250 ± 20% respectively, with these levels of tongue muscle activity after CNO reaching 93 ± 12% and 58 ± 8% of those measured during quiet wakefulness after the vehicle control in the same time period (Fig. 7). This lesser response in REM sleep likely reflects the endogenous changes in sleep-dependent neuromodulators occurring at the hypoglossal motor pool^12^,^16^,^27^ that, in the context of human studies, any pharmacotherapy must overcome in order to maintain sufficient neuromuscular activity for adequate airflow and ventilation^29^,^30^,^31^,^32^,^33^. Nevertheless, sustained tongue muscle activation was still observed throughout REM sleep in response to CNO (Figs 5, 7 and 9).

It is also important to note that although the tongue muscle activation produced by CNO was sustained (Fig. 9) and specific to the tongue musculature (Fig. 8), it was physiological in pattern (i.e., tonic activation) and reached a magnitude not appreciably different from that normally experienced in quiet wakefulness (Figs 5, 7 and 9). The data also show that there were no effects of CNO on breathing (as judged by diaphragm recordings), postural muscle tone (as judged by neck EMG recordings), or brain arousal state (as judged by EEG activity). Figure 1 indicates that there was effective transduction of the viral vector, as shown by mCherry expression at the hypoglossal motor nucleus, and that it was reasonably restricted to this nucleus within and between mice. Nevertheless, we cannot exclude that there were changes in other parameters that were not specifically measured such as the magnitude of tongue muscle activation during particular behaviors such as swallowing, licking, grooming, or vocalization. Nor can we exclude changes in other motor or autonomic functions mediated by nearby local circuits that may have been impacted by ‘spillover’ of the DREADD beyond the hypoglossal motor pool.

The systemically applied CNO produced sustained (8–10 hours) tongue muscle activation, but this activation returned to normal baseline values each day (Figs 3 and 9). This result indicates that the activation was not the result of irreversible changes in the mechanisms controlling hypoglossal motor activity. Indeed, previous studies modulating other discrete cell populations transduced with hM3Dq receptors or other DREADDs have reported effects that also persist for similar durations^34^,^35^,^36^.

### Use of DREADDs and mechanism of action

Here we implemented the DREADDs technology that uses bioengineered G protein-coupled receptors to control intracellular signalling pathways in select cell populations^19^,^37^. In this case we selectively manipulated cholinergic neurons at the hypoglossal motor pool by using targeted introduction of viral vectors in ChAT-Cre^+^ mice. The hM3Dq DREADDs are type 3 human muscarinic acetylcholine receptors (hM3) mutated through a process of directed molecular evolution in yeast^19^. The hM3 DREADD (hM3D) contains a mutation in the third and fifth trans-membrane domains, resulting in a loss of affinity for acetylcholine, and a nano-molar sensitivity for the blood brain barrier diffusible compound CNO which is otherwise biologically inert^17^,^37^. The association of the hM3 receptors with Gαq subunits (hM3Dq) produces neuronal excitation when activated by the exogenous ligand CNO^19^,^37^.

The mechanism of action of CNO is thought to involve hM3Dq-induced stimulation of membrane bound phospholipase C beta (PLC-β) which then causes hydrolysis of phosphatidylinositol bisphosphate (PIP_2_) into inositol triphosphate (IP_3_) and diacylglycerol (DAG); DAG then activates protein kinase C. The IP_3_ can trigger calcium release from the endoplasmic reticulum to activate voltage-dependent calcium channels and contribute to neural depolarization^19^,^37^. Of particular relevance to the present study is that Kir channels require the docking of PIP_2_ for their activity^38^,^39^, such that CNO-induced hydrolysis of PIP_2_ increases neuronal excitability through inactivation of such constitutively active potassium channels^19^,^37^. Accordingly, the activation of the “designer” receptors in the present study may have produced the excitation of the hypoglossal motor pool, in part by downstream effects that influenced the Kir channels that are ultimately the desired target for pharmacological development^13^,^16^.

Overall, the findings of the present study show that local incorporation of the viral vector that incorporated the DREADDs (hM3Dq) transgene led to significant, selective and sustained tongue muscle activation in response to systemic application of the ligand CNO. No response to CNO was obtained in the mice in which the hypoglossal motor pool was transduced with the viral vector that lacked the DREADDs (hM3Dq) transgene, i.e., the inactive virus controls. These findings and the above discussion serve to highlight that hypoglossal motoneurons contain the intracellular machinery necessary to produce neuronal activation in response to hM3Dq activation by the *systemically* administered CNO.

### Modulation of the tongue motor activity

The hypoglossal motor nucleus innervates both the extrinsic and intrinsic muscles of the tongue. The intrinsic muscles have their origin and insertion arising from the inside of the tongue, and are comprised of the superior and inferior longitudinal, and the traverses and verticals linguae. The extrinsic muscles have their origin and insertions arising from outside of the tongue, and comprise the genioglossus, palatoglossus, hyoglossus and styloglossus. Some of the extrinsic muscles of the tongue have classically been designated as tongue “protruders” or “retractors” based on the original anatomical studies, with the intrinsic muscles being more classically associated with changes in tongue shape. However, co-activation of the intrinsic and extrinsic muscles of the tongue (i.e., including both “retractors” and “protruders”) contributes to increases in upper airway stability and resistance to airway closure in animals and humans^40^,^41^,^42^,^43^,^44^. Accordingly, targeted activation of hypoglossal motoneurons and their innervated muscles *en masse* (i.e., rather than targeted activation of a particular or singular muscle group) would be expected to have net positive benefits to upper airway stability and OSA.

Given the expression of the viral vector containing the DREADDs transgene (hM3Dq) occurs throughout the hypoglossal motor pool (Fig. 1), the CNO administered in the present study would also be expected to activate the tongue musculature *en masse*. Selective recordings from individual muscle groups is not possible given the small size of the mouse tongue, as illustrated by the inherent variation in electrode placements and size of the electrode tips (Fig. 4). However, the *in*-*vivo* recordings of the present study do identify that the systemically administered CNO produced robust, selective and sustained (i.e., for hours) activation of the tongue musculature during sleep that subsequently returned to baseline levels, with no off-target effects as evidenced by the absence of changes in other physiological variables.

## Additional Information

**How to cite this article:** Horton, G. A. *et al*. Activation of the Hypoglossal to Tongue Musculature Motor Pathway by Remote Control. *Sci. Rep.* **7**, 45860; doi: 10.1038/srep45860 (2017).

**Publisher's note:** Springer Nature remains neutral with regard to jurisdictional claims in published maps and institutional affiliations.

## References

[b1] Remmers J. E., deGroot W. J., Sauerland E. K. & Anch A. M. Pathogenesis of upper airway occlusion during sleep. J Appl Physiol 44, 931–938 (1978). 670014 10.1152/jappl.1978.44.6.931

[b2] Phillipson E. A. Sleep apnea - a major public health problem. New Eng J Med 328, 1271–1273 (1993). 8464440 10.1056/NEJM199304293281712

[b3] Young T., Peppard P. E. & Gottlieb D. J. Epidemiology of obstructive sleep apnea: a population health perspective. Am J Respir Crit Care Med 165, 1217–1239 (2002). 11991871 10.1164/rccm.2109080

[b4] Malhotra A. & White D. P. Obstructive sleep apnoea. Lancet 360, 237–245 (2002). 12133673 10.1016/S0140-6736(02)09464-3

[b5] Buchanan P. & Gruntstein R. In Principles and Practice of Sleep Medicine (eds Kryger M. H., Roth T. & Dement W. C.) 1233–1249 (Elsevier, Saunders, 2011).

[b6] Atwood C. W., Strollo P. J. & Givelber R. In Principles and Practice of Sleep Medicine (eds Kryger M. H., Roth T. & Dement W. C.) 1219–1232 (Elsevier, Saunders, 2011).

[b7] Eastwood P. R. . Obstructive Sleep Apnoea: From pathogenesis to treatment: Current controversies and future directions. Respirology 15, 587–595 (2010). 20136736 10.1111/j.1440-1843.2009.01699.x PMC4342116

[b8] Smith I., Lasserson T. J. & Wright J. Drug therapy for obstructive sleep apnoea in adults. The Cochrane database of systematic reviews CD003002 (2006). 16625567 10.1002/14651858.CD003002.pub2

[b9] Smith I. E. & Quinnell T. G. Pharmacotherapies for obstructive sleep apnoea: where are we now? Drugs 64, 1385–1399 (2004). 15212557 10.2165/00003495-200464130-00001

[b10] Kohler M., Bloch K. E. & Stradling J. R. Pharmacological approaches to the treatment of obstructive sleep apnoea. Expert Opin Investig Drugs 18, 647–656 (2009). 10.1517/13543780902877674 19388881

[b11] Kohler M. & Stradling J. R. Pitfalls of clinical trials on pharmacological treatment for obstructive sleep apnoea: future directions. Expert Opin Investig Drugs, doi: 10.1517/13543784.2011.590473 (2011). 21635196

[b12] Grace K. P., Hughes S. W. & Horner R. L. Identification of the mechanism mediating genioglossus muscle suppression in REM sleep. Am J Respir Crit Care Med 187, 311–319 (2013). 23220910 10.1164/rccm.201209-1654OC

[b13] Grace K. P., Hughes S. W. & Horner R. L. Identification of a pharmacological target for genioglossus reactivation throughout sleep. Sleep 37, 41–50, doi: 10.5665/sleep.3304 (2014). 24470694 PMC3902869

[b14] Töpert C. . Kir2.4: a novel K+ inward rectifier channel associated with motoneurons of cranial nerve nuclei. J Neurosci 18, 4096–4105 (1998). 9592090 10.1523/JNEUROSCI.18-11-04096.1998 PMC6792806

[b15] Lein E. S. . Genome-wide atlas of gene expression in the adult mouse brain. Nature 445, 168–176 (2007). 17151600 10.1038/nature05453

[b16] Grace K. P., Hughes S. W., Shahabi S. & Horner R. L. K+ channel modulation causes genioglossus inhibition in REM sleep and is a strategy for reactivation. Respir Physiol Neurobiol 188, 277–288, doi: 10.1016/j.resp.2013.07.011 (2013). 23872455

[b17] Armbruster B. N., Li X., Pausch M. H., Herlitze S. & Roth B. L. Evolving the lock to fit the key to create a family of G protein-coupled receptors potently activated by an inert ligand. Proc Natl Acad Sci USA 104, 5163–5168, doi: 10.1073/pnas.0700293104 (2007). 17360345 PMC1829280

[b18] Dong S., Rogan S. C. & Roth B. L. Directed molecular evolution of DREADDs: a generic approach to creating next-generation RASSLs. Nat Protoc 5, 561–573, doi: 10.1038/nprot.2009.239 (2010). 20203671

[b19] Rogan S. C. & Roth B. L. Remote control of neuronal signaling. Pharmacol Rev 63, 291–315, doi: 10.1124/pr.110.003020 (2011). 21415127 PMC3082452

[b20] Wu Z., Asokan A. & Samulski R. J. Adeno-associated virus serotypes: vector toolkit for human gene therapy. Mol Ther 14, 316–327, doi: 10.1016/j.ymthe.2006.05.009 (2006). 16824801

[b21] Grace K. P., Liu H. & Horner R. L. 5-HT1A receptor-responsive pedunculopontine tegmental neurons suppress REM sleep and respiratory motor activity. J Neurosci 32, 1622–1633 (2012). 22302804 10.1523/JNEUROSCI.5700-10.2012 PMC6703359

[b22] Mesbah-Oskui L., Orser B. A. & Horner R. L. Thalamic delta-subunit containing GABAA receptors promote electrocortical signatures of deep non-REM sleep but do not mediate the effects of etomidate at the thalamus *in vivo*. J Neurosci 34, 12253–12266, doi: 10.1523/jneurosci.0618-14.2014 (2014). 25209268 PMC6615504

[b23] Montandon G. . G-protein-gated inwardly rectifying potassium channels modulate respiratory depression by opioids. Anesthesiology 124, 641–650, doi: 10.1097/aln.0000000000000984 (2016). 26675532 PMC4755838

[b24] Franklin K. B. J. & Paxinos G. The mouse brain in stereotaxic coordinates. 3rd edn (New York, 2008).

[b25] Saper C. B. & Scammell T. E. Emerging therapeutics in sleep. Ann Neurol 74, 435–440, doi: 10.1002/ana.24000 (2013). 24038193

[b26] Bartus R. T. . Safety/feasibility of targeting the substantia nigra with AAV2-neurturin in Parkinson patients. Neurology 80, 1698–1701, doi: 10.1212/WNL.0b013e3182904faa (2013). 23576625 PMC3716474

[b27] Chan E., Steenland H. W., Liu H. & Horner R. L. Endogenous excitatory drive modulating respiratory muscle activity across sleep-wake states. Am J Respir Crit Care Med 174, 1264–1273 (2006). 16931636 10.1164/rccm.200605-597OC

[b28] Taranto-Montemurro L. . Desipramine increases genioglossus activity and reduces upper airway collapsibility during non-REM sleep in healthy subjects. Am J Respir Crit Care Med, doi: 10.1164/rccm.201511-2172OC (2016). PMC5074653 26967681

[b29] Patil S. P. . Neuromechanical control of upper airway patency during sleep. J Appl Physiol 102, 547–556 (2007). 17008440 10.1152/japplphysiol.00282.2006

[b30] Younes M. Contributions of upper airway mechanics and control mechanisms to severity of obstructive apnea. Am J Respir Crit Care Med 168, 645–658 (2003). 12773321 10.1164/rccm.200302-201OC

[b31] Younes M. Role of respiratory control mechanisms in the pathogenesis of obstructive sleep disorders. J Appl Physiol 105, 1389–1405 (2008). 18787092 10.1152/japplphysiol.90408.2008

[b32] Eckert D. J., White D. P., Jordan A. S., Malhotra A. & Wellman A. Defining phenotypic causes of obstructive sleep apnea: identification of novel therapeutic targets. Am J Respir Crit Care Med in press, doi: 10.1164/rccm.201303-0448OC (2013). PMC3826282 23721582

[b33] Carberry J. C., Jordan A. S., White D. P., Wellman A. & Eckert D. J. Upper Airway Collapsibility (Pcrit) and Pharyngeal Dilator Muscle Activity are Sleep Stage Dependent. Sleep 39, 511–521, doi: 10.5665/sleep.5516 (2016). 26612386 PMC4763361

[b34] Alexander G. M. . Remote control of neuronal activity in transgenic mice expressing evolved G protein-coupled receptors. Neuron 63, 27–39, doi: 10.1016/j.neuron.2009.06.014 (2009). 19607790 PMC2751885

[b35] Krashes M. J. . Rapid, reversible activation of AgRP neurons drives feeding behavior in mice. J Clin Invest 121, 1424–1428, doi: 10.1172/jci46229 (2011). 21364278 PMC3069789

[b36] Farrell M. S. & Roth B. L. Pharmacosynthetics: Reimagining the pharmacogenetic approach. Brain Res 1511, 6–20, doi: 10.1016/j.brainres.2012.09.043 (2013). 23063887 PMC3562395

[b37] Urban D. J. & Roth B. L. DREADDs (designer receptors exclusively activated by designer drugs): chemogenetic tools with therapeutic utility. Annu Rev Pharmacol Toxicol 55, 399–417, doi: 10.1146/annurev-pharmtox-010814-124803 (2015). 25292433

[b38] Hansen S. B., Tao X. & MacKinnon R. Structural basis of PIP2 activation of the classical inward rectifier K+ channel Kir2.2. Nature 477, 495–498, doi: 10.1038/nature10370 (2011). 21874019 PMC3324908

[b39] Soom M. . Multiple PIP2 binding sites in Kir2.1 inwardly rectifying potassium channels. FEBS Lett 490, 49–53 (2001). 11172809 10.1016/s0014-5793(01)02136-6

[b40] Bailey E. F. & Fregosi R. F. Coordination of intrinsic and extrinsic tongue muscles during spontaneous breathing in the rat. J Appl Physiol 96, 440–449 (2004). 14527967 10.1152/japplphysiol.00733.2003

[b41] Bailey E. F., Huang Y. H. & Fregosi R. F. Anatomic consequences of intrinsic tongue muscle activation. J Appl Physiol 101, 1377–1385 (2006). 16825524 10.1152/japplphysiol.00379.2006

[b42] Fregosi R. F. Influence of tongue muscle contraction and dynamic airway pressure on velopharyngeal volume in the rat. J Appl Physiol 104, 682–693 (2008). 18079270 10.1152/japplphysiol.01043.2007

[b43] Fuller D. D., Williams J. S., Janssen P. L. & Fregosi R. F. Effect of co-activation of tongue protrudor and retractor muscles on tongue movements and pharyngeal airflow mechanics in the rat. J Physiol 519, 601–613 (1999). 10457075 10.1111/j.1469-7793.1999.0601m.x PMC2269504

[b44] Mateika J. H., Millrood D. L., Kim J., Rodriguez H. P. & Samara G. J. Response of human tongue protrudor and retractors to hypoxia and hypercapnia. Am J Respir Crit Care Med 160, 1976–1982 (1999). 10588616 10.1164/ajrccm.160.6.9903001

